# The double burden of under- and overnutrition among Bangladeshi women: Socioeconomic and community-level inequalities

**DOI:** 10.1371/journal.pone.0219968

**Published:** 2019-07-25

**Authors:** Md. Aminur Rahman, Md. Mosfequr Rahman, Md. Mosiur Rahman, Syeda S. Jesmin

**Affiliations:** 1 Department of Population Science and Human Resource Development, University of Rajshahi, Rajshahi, Bangladesh; 2 Department of Global Health Entrepreneurship, Division of Public Health, Tokyo Medical and Dental University, Tokyo, Japan; 3 Sociology and Psychology Department, University of North Texas at Dallas, Dallas, Texas, United States of America; Anglia Ruskin University, UNITED KINGDOM

## Abstract

**Background:**

The prevalence of overweightness in Bangladesh is increasing, while underweightness also continues to persist. A better understanding of the patterns and socioeconomic risk factors of both conditions, particularly among women, is critical in order to promote the development of interventions to improve maternal health in Bangladesh. This study therefore sought to assess the patterns of under- and overweightness between 2004 and 2014 and to examine the predictors of individual and community-level inequalities of under- and overnutrition in Bangladesh.

**Methods:**

Cross-sectional data of 10, 431, and 16,478 ever-married nonpregnant women aged between 15 and 49 years who did not give birth in the two months preceding the survey were extracted from the 2004 and 2014 Bangladesh Demographic and Health Surveys, respectively. Body mass index was used to measure weight status, and underweightness, at-risk for overweightness, overweightness, and obesity were the main outcome variables. Patterns of nutritional change over time was examined by considering the annual average rate of change. Multilevel multinomial logistic regression and quantile regression were used to identify the inequalities.

**Results:**

In 2014, the age-adjusted prevalence values of underweightness, at-risk for overweightness, overweightness, and obesity were 19.7%, 14.9%, 18.1% and 4.0%, respectively. A higher average annual rate of reduction of underweightness was found among wealthier, highly educated, and wealthier community–living women, while a rate of increase of overweightness was found among poorer, uneducated, and poor community–living women. Individual and community-level inequalities of malnutrition were observed among these populations. In comparison with women living in low wealth communities, women from wealthier communities were at an increased risk of being at-risk for overweightness [adjusted odds ratio (AOR): 1.53, 95% confidence interval (CI): 1.23–1.91], overweight (AOR: 1.60, 95% CI: 1.27–2.00), and obese (AOR: 2.12, 95% CI: 1.42–3.18).

**Conclusions:**

This study suggests the coexistence of a double burden of under- and overnutrition in Bangladesh and that the prevalence of overweightness surpasses that of underweightness. The burdens of under- and overnutrition are strongly associated with women’s individual socioeconomic positions and the nature of the community in which they live.

## Introduction

Many developing countries are currently facing the paradoxical coexistence of under- and overnutrition—known as the double burden of nutrition [1]—which could be attributed to their rapidly growing economy, ongoing demographic changes, and continued urbanization [2, 3]. This phenomenon may also be attributed to changing dietary patterns of the people, especially with respect to the increasing consumption of processed and ultra-processed food [4]. These nutritional conditions constitute a major public health concern, since both under- and overnutrition are among the top 10 leading risk factors for the global burden of diseases [5]. The prevalence rates of underweightness and overweightness among reproductive-aged women are of particular interest because both of these conditions are not only detrimental to women’s health but the health of their children. For example, a number of studies have documented that being underweight might reduce a woman’s fertility while increasing her risks of adverse pregnancy outcomes such as low birth weight, small for gestational age size, preterm birth, and neonatal death [6, 7]. On the other hand, overweight and obese women bear higher risks for infertility and gestational complications such as hypertensive disorder, gestational diabetes, haemorrhage, and caesarean delivery as well as higher risk factors for foetal and infant death, neural tube defects, and newborn macrosomia [8, 9]. Maternal overweightness and obesity also have intergenerational effects: for example, the children of obese women are at an increased risk of being obese during childhood and in early adulthood and also have a higher risk of suffering from diabetes and cardiovascular diseases later in life [10]. Therefore, it is imperative to understand the extent and distribution of both types of malnutrition and their determinants, especially in low-income poor settings, so as to channel the public health resources appropriately.

Nutrition transition refers to predictable shifts in dietary pattern due to rapid urbanization, modernization, economic development, and increased wealth [11, 12], and Bangladesh is going through this nutritional transition [13]. Therefore, the prevalence of overweightness and obesity is rapidly increasing, while the prevalence of underweightness continues to persist in Bangladesh [14]. The most recent nationally representative health survey report of Bangladesh showed that the percentages of underweightness and overweightness among reproductive-aged women were 19% and 24%, respectively [15]. To date, the undernutrition aspect of malnutrition is the prioritized area in almost all policies of the government of Bangladesh, while the problem of overnutrition, the other form of malnutrition, does not receive as much attention. However, the prevalence of overweightness among women in Bangladesh has increased considerably over the last decade. Therefore, in order to control overnutrition, health and nutrition policies need to be revised. To formulate appropriate policies, it is crucial to identify the determinants that have substantial effects on malnutrition—both undernutrition and overnutrition—among reproductive-aged women.

Certain individual and socioeconomic factors have already been identified to be associated with a woman being under- oroverweight in several developing countries [16–19] including Bangladesh [14, 20–22]. A recent meta-analytical review of Bangladesh observed an annual average rate of a reduction of underweightness occurred among women with a higher socioeconomic status, higher education, and urban living status, while a higher annual average rate of an increase of overweightness was found among women with a lower socioeconomic status, lower level of literacy, and rural living status [23]. The shift from low body mass index (BMI; ratio of weight in kilograms to the square of height in meters) to high BMI was positively associated with urban residence, age, higher socioeconomic status, and higher education. Besides various individual-level variables, the nature of the community where the women live might have a significant impact on their probability of being under- or overweight. For example, Corsi et al. [24] documented that community wealth exerts an important influence on being malnourished, and suggested that community wealth was positively associated with overnutrition and negatively with undernutrition. However, they drew their conclusions using older data from the 2004 Bangladesh Demographic and Health Survey (BDHS), when there was a lack of coexistence of underweightness and overweightness. Moreover, they limited their research to considering community wealth only and ignored some other community-level variables such as education or media access. An individual’s education was found to have substantial effects on the nutritional status of women [24, 25], but how community-level illiteracy status affects the nutritional status of women was not examined in their study.

Over the last few decades, in changing various health behaviours in mass populations, media campaigns (e.g., radio, television, newspaper) have played an important role in both developing and developed countries [26, 27]. Mass media has a potential impact on the acquisition of proper maternal health care services in developing countries [28, 29], which might play a vital role in women receiving proper nutritional knowledge as well. Although media serves as the most popular source of nutrition information for the public [30], no explicit study was found that assessed the relationship between mass media access and nutritional status. It is expected that community-level illiteracy status and access to media might have substantial roles in women’s nutritional status. In particular, women from more literate communities and those that have more media access are generally better aware of how to utilize available resources for the improvement of their own health status as well as that of their families. Therefore, besides education, socioeconomic status, and community-wealth status, we sought to identify how nutritional inequalities exist in conjunction with an individual’s media access and community-illiteracy status. We also wanted to elucidate the change in nutritional status over last 10 years among women in Bangladesh. Therefore, using the most recent nationally representative data from Bangladesh, the specific objectives of this study were (1) to investigate the patterns of under- and overweightness among women aged 15 to 49 years from 2004 to 2014 and (2) to examine the changes in the weight status of women by individual sociodemographic and community-level variables.

## Methods

### Data source

**Fig 1:**
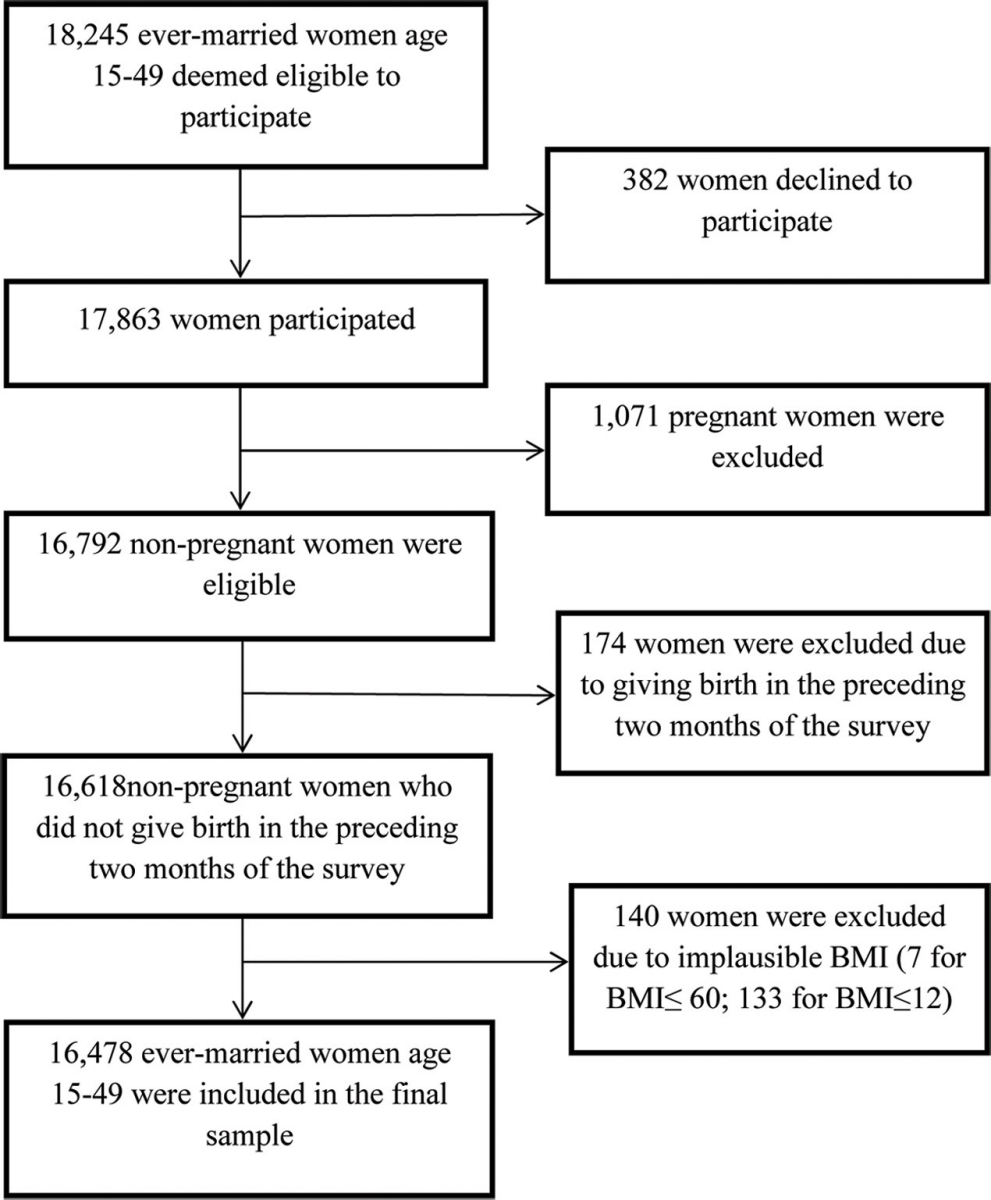
Flowchart showing the selection procedure of the sample from the 2014 BDHS.

Data for this study were based on the most recent nationally representative and cross-sectional round of the 2014 Bangladesh Demographic and Health Survey (BDHS).The BDHS is periodically conducted by the National Institute of Population Research and Training, Bangladesh in collaboration with the United States Agency for International Development and ICF International (Fairfax, VA, USA). A multistage cluster sampling design was used to collect data on fertility; mortality; family planning; and various important aspects of nutrition, health, and health care. The BDHS used standard model questionnaires that were designed for and widely used in developing countries [31]. Primary sampling units (PSUs) for the 2014 BDHS were based on enumeration areas from the national population and housing census conducted in 2011 by the Bangladesh Bureau of Statistics (BBS) and the covered entire country. About 600 PSUs (207 from urban areas and 393 from rural areas) from each of Bangladesh’s administrative divisions were selected first, with probability proportional to the unit size. A systematic sample of 30 households on average was selected per unit in the second stage. Detailed information about the surveys and other related issues of the BDHS is available elsewhere [15]. All ever-married women aged 15 to 49 years in the selected households who were not pregnant and who did not give birth in the two months preceding the survey were eligible to be included in the final sample. Although there is no single definition of what constitutes an implausible value, using criteria indicated by a demographic and health survey methodological report [32], we excluded extreme BMI values (≤ 12 kg/m^2^ or ≥ 60 kg/m^2^) from the sample, leading to the assembly of a total sample of 16,478 women. Fig 1 details the procedure of study sample selection. We also used data from the 2004 BDHS in some part of the analysis. With the same eligibility criteria, the sample size for the 2004 BDHS was 10,431 women.

### Outcomes

BMI was used to classify the nutritional status of the sample and was used as the outcome measure for this study. Following the World Health Organization conventions appropriate for Asian populations [33], the BMI cutoff points adopted in this study were as follows: less than 18.5kg/m^2^ (underweight), 18.5 to 22.9kg/m^2^ (normal weight), 23 to 24.9kg/m^2^ (at-risk for overweightness), 25 to 29.9kg/m^2^ (overweight), and 30kg/m^2^or more (obese). The “normal” BMI range of 18.5 to 24.9 kg/m^2^ was narrowed to 18.5 to 22.9 kg/m^2^ because, in Asian populations [33], a BMI of 23 kg/m^2^ is identified as a public health cutoff point for the risk of obesity, and earlier evidence as suggested that lower cutoff points are appropriate for populations of the Indian subcontinent [34, 35]. Additionally, in accordance with earlier studies in India [19, 25, 36] and Bangladesh [14], individuals with BMIs of between 23 and 24.9kg/m^2^ are categorized as “at-risk for overweightness” because some data across the world [37–39], particularly in Asia [33, 40], have suggested that the risk of cardiovascular disease and all-cause mortality increases with BMI values of lower than 25kg/m^2^.

### Exposures

Three measures of socioeconomic status were employed in this study: household wealth, education, and media access. Wealth was defined in terms of household assets and material possessions. Each woman was assigned a household wealth score based on a linear combination of the scores for different households’ assets ownership, dwelling amenities, and living conditions, which were weighted according to a principal components analysis [41]. The resulting wealth score variable was standardized to have a mean value of zero and a standard deviation value of one [42]. The weighted scores were divided into quintiles and these quintile cutoffs were survey time–dependent. Women’s educations were measured according to significant milestones in the formal Bangladeshi education system that were adopted as cutoff points: zero (none), one to five years (primary), six to 10 years (secondary) and 11 years or more (higher). An additive media access index was created that measured the frequency with which a woman was exposed to any of the following three media types: television, radio, and newspapers/magazines. The additive media access index was composed of values ranging from zero to six points, where zero points indicated no media access at all and six points indicating using the three media types at least once a week. Finally, women were divided into tertiles of media access, as follows: low, medium, and high.

We created two measures of community-level variables: community wealth status and community illiteracy status. According to the 2014 BDHS, an individual’s illiteracy is defined as the person’s inability to read all or part of a sentence. Individual (illiteracy) and household-level (household wealth) variables were aggregated at the level of PSU to create the community-level illiteracy and community-level wealth variables. The generated community-level variables were divided into three tertiles and categorized as low, medium, or high.

### Covariates

This study considers a range of individual demographic and socioeconomic covariates on the basis of existing literature and the availability of relevant data. Age was categorized into five-year groups ranging in total from 15 to 49 years. Since only ever-married women were interviewed in the BDHS, marital status was classified as either married, widowed, or separated. Respondents’ religion was categorized as either Muslim or non-Muslim, since Bangladesh is predominantly a Muslim country [15]. Occupation of the respondent was defined as either non-manual work (e.g. professional/technical/managerial, sales, services), manual work (e.g. paid household or domestic, skilled and unskilled manual), agricultural work (agricultural-self-employed or employee), or homemaker (not participating in the labour force). Current contraception use was categorized as not using, oral contraception, or other (e.g., injections, condom, withdrawal, periodic abstinence). Decision-making power in household index was measured based on responses to individual questions regarding who makes decisions in the (respondent’s) household regarding the following: (1) obtaining health care; (2) large household purchases; and (3) visits to family or relatives. The response options were: (a) respondent alone, (b) respondent and husband/partner, (c) respondent and other person, (d) husband/partner alone, (e) someone else, and (f) other. For each question, one point was assigned if the response was (a), (b), or (c), while zero points were assigned for an answer of (d), (e), or (f). The values were then added, resulting in a score ranging from zero to three points (Cronbach α = 0.78). Other covariates include whether children ever born, place of residence (rural or urban), and region (Barisal, Chittagong, Dhaka, Khulna, Rajshahi, Rangpur, Sylhet).

### Statistical analysis

All statistical analyses of this study were done in Stata 13.1/MP (StataCorp LLC, College Station, TX, USA). Using the national weights allocated by the BDHS survey design, the weighted prevalence values of women who were underweight, at-risk for overweightness, overweight, and obese within each of the predictor variables were calculated. The age-standardized prevalence values of underweightness, at-risk for overweightness, overweightness, and obesity were additionally calculated using the 2011 national population and housing census. Standard errors were generated on the basis of the Taylor series linearization method. We used the chi-squared test to compare the differences in prevalence between groups with different individual, sociodemographic, and community-level variables. To assess the rate of change of the outcomes variables (i.e., underweightness, at-risk for overweightness, overweightness, and obesity) in last 10 years, we calculated the average annual rate of change including the annual average rate of increase (AARI) and annual average rate of reduction (AARR) between the 2004 and 2014 BDHSs. The annual rate of change was calculated via the equation Y_t+n_ = Y_t_*(1±b%)^n^[43], where Y_t_ = the prevalence of the four weight categories in 2004, b = the annual rate of change, n = the number of years between the 2004 and 2014 BDHSs, and Y_t+n_ = the prevalence of the four weight categories in 2014.

To fulfil the objective of our study, we used the following two different sets of major analyses: multinomial logistic regression and quantile regression. The 2014 BDHS dataset was based on multistage stratified cluster sampling. The appropriate approach to analysing BMI data from this survey was, therefore, based on nested sources of variability such as individuals who were nested within community. To account for the hierarchical data structure of the BDHS, we used a two-level multinomial regression to predict the nutritional status of women using a generalized linear mixed model with a logit link-function [44]. Normal BMI (18.5–22.9 kg/m^2^) was taken as the reference outcome category in the multinomial model.

Due to the arbitrariness of the cutoff points, multinomial outcome modelling can suffer from the loss of information. Therefore, to overcome such a problem, in a second set of analyses, we specified a quantile regression model, which used the continuous representation of BMI as the outcome variable [45, 46].Moreover, this nonparametric quantile regression model technique is relatively robust to the influence of outliers because, in this technique, neither specific distributional assumptions nor homoscedasticity are assumed for the error term. Quantile regression could be used to identify more vulnerable groups and devise more effective interventions because it provides a more complete or better presentation of the effect of an independent variable on the outcome variable. For countries where both underweight and overweight data are critical, the conditional distribution of BMI is certainly of particular interest for improving the health of the general public. Quantile regression explores the marginal effects of the predictor variables on the entire distribution of the dependent variable (BMI in the current study). In this study, the 10^th^, 25^th^, 50^th^, 75^th^, and 90^th^ BMI quantiles were specified in the model. Standard errors and confidence intervals for the quantile regression coefficient estimates were obtained using 1,000 bootstrap replications.

### Ethical considerations

We obtained the data used in this study from the MEASURE DHS archive. The data were originally collected by ICF International (Fairfax, VA, USA) and followed a standard protocol approved by the ICF International Review Board. The BDHS also received approval from the National Ethics Committee in Bangladesh. Informed consent was obtained from each respondent of this survey. This study was considered to be exempt, however, from full review, as it was based on an anonymous public use of a secondary dataset with no identifiable information on the survey participants.

## Results

**Fig 2:**
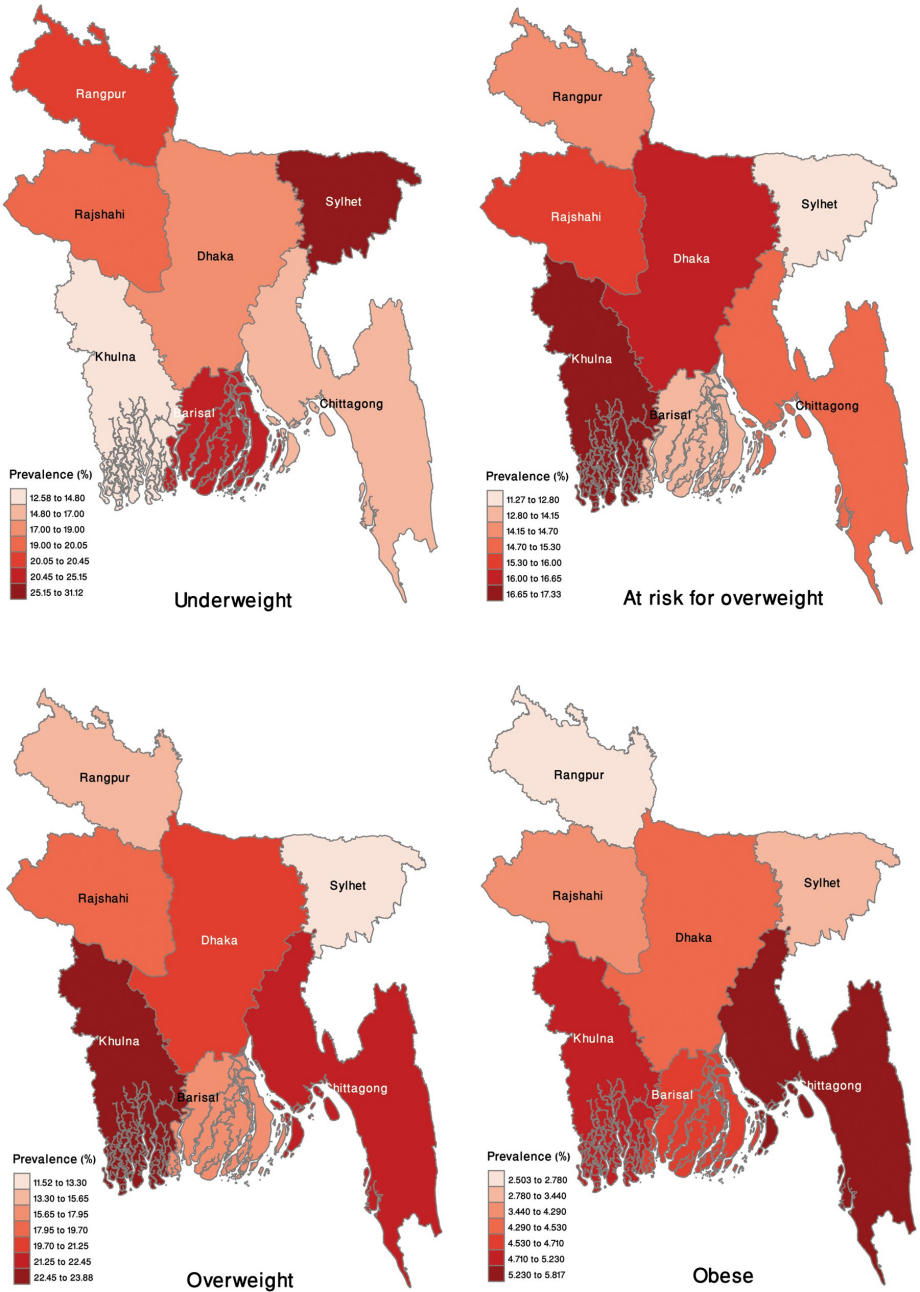
Regional distribution of the four weight categories among reproductive-aged women in Bangladesh in 2014.

The age-adjusted prevalences of different malnutrition categories are presented in Table 1, according to the individual- and community-level characteristics. Overall, the age-adjusted prevalence values of underweightness, at-risk for overweightness, overweightness, and obesity were 19.7% [95% confidence interval (CI): 18.5%–20.8%], 14.9% (95% CI: 14.1%–15.7%), 18.1% (95% CI: 17.0%–19.2%), and 4.0% (95% CI: 3.5%–4.6%), respectively. The age-adjusted prevalence of underweightness among women with no education was more than three times that in women with higher education (28.3% vs. 8%). In contrast, the age-adjusted prevalence values of being at-risk for overweightness (12.2% vs. 18.4%), overweightness (10.1%% vs. 32.5%), and obesity (1.7% vs. 8.7%) were lower among women with no education versus their counterparts with higher education. The prevalence of underweightness was high among women of the bottom quintile for household wealth, in those with low media access, and in those who were living in a low-wealth, high-illiteracy community as compared with those in the top-household-wealth quintile with high media access and a high-wealth, low-illiterate community, respectively. On the other hand, the prevalences of being at-risk for overweightness, overweightness, and obesity were higher among women of latter group. However, the age-adjusted prevalence of underweightness among rural residents was nearly double that in urban residents at 13.5% versus 22.1%. In contrast, the age-adjusted prevalence values of being at-risk for overweightness, overweightness, and obesity were much lower among rural versus urban women. The geographic patterns of unadjusted prevalences of underweightness, at-risk for overweightness, overweightness, and obesity are presented in Fig 2. The prevalence of underweightness was higher among women in the Sylhet division, while being at-risk for overweightness and actually overweight were higher in the Khulna division. However, a higher prevalence of obesity among women was observed in Chittagong (Fig 2).

**Table 1:** Age-adjusted prevalence of underweight, at risk for overweight, overweight, and obesity for women aged 15–49 years (n = 16,478) by individual socio-demographic and community-level variables: Bangladesh Demographic Health Survey, 2014.

	Underweight BMI <18.5 (n = 3069)			At risk for overweight BMI = 23.0–24.9(n = 2538)			Overweight BMI = 25.0–29.9 (n = 3193)				Obese BMI ≥30.0 (n = 722)		
		95% CI			95% CI			95% CI				95% CI	
Variables	%	low	high	%	low	high	%		low	high	%	low	high
Overall prevalence	19.7	18.5	20.8	14.9	14.1	15.7	18.1		17.0	19.2	4.0	3.5	4.6
**Marital status**													
Married	19.2	18.1	20.3	15.1	14.3	15.9	18.5		17.4	19.7	4.1	3.6	4.7
Widowed	24.6	14.2	34.9	15.7	6.8	24.6	13.7		5.2	22.2	2.3	0.7	3.9
Divorced/separated	26.4	21.1	31.7	9.9	6.6	13.2	9.8		6.7	12.9	3.3	1.3	5.4
*P*-value	0.001			0.053			<0.001				0.878		
**Religion**													
Non-Muslim	17.9	14.9	20.9	14.6	11.8	17.4	17.7		14.2	21.1	4.6	2.8	6.5
Muslim	20.0	18.6	21.1	15.0	14.1	15.8	18.1		17.0	19.3	4.0	3.4	4.8
*P*-value	0.112			0.804			0.755				0.314		
**Education**													
None	28.3	25.0	31.8	12.2	10.2	14.2	10.1		8.4	11.8	1.7	1.2	2.3
Primary	22.5	20.1	25.0	13.8	12.4	15.1	15.3		14.2	16.4	3.2	2.7	3.8
Secondary	14.4	13.1	15.7	16.5	15.3	17.8	24.1		22.6	25.7	6.6	5.4	7.7
Higher	8.1	6.4	9.8	18.4	16.1	20.6	32.5		29.6	35.3	8.7	7.0	10.4
*P*-value	<0.001			0.013			<0.001				<0.001		
**Employment status**													
Homemaker	19.1	17.8	20.3	15.3	14.4	16.1	20.0		18.8	21.3	5.1	4.3	5.8
Agricultural	22.9	19.9	26.0	12.8	10.9	14.7	12.0		10.3	13.7	1.6	0.9	2.2
Non-manual	14.2	11.5	17.0	14.9	12.5	17.3	19.1		16.4	21.7	2.8	1.9	3.8
Manual	20.6	17.3	24.0	14.1	11.9	16.3	17.3		15.1	19.5	3.0	2.1	3.9
*P*-value	0.003			0.806			<0.001				<0.001		
**Household wealth**													
Bottom quintile	33.2	30.1	36.2	9.0	7.5	10.5	6.9		5.6	8.2	0.8	0.4	1.2
Second quintile	26.1	23.8	28.5	13.4	11.8	14.9	11.3		9.8	12.8	1.0	0.6	1.4
Third quintile	19.8	18.0	21.6	14.1	12.2	16.0	16.6		14.8	18.3	2.6	1.8	3.3
Fourth quintile	13.3	11.7	14.9	19.4	16.8	22.1	21.3		19.5	23.1	4.5	3.7	5.2
Top quintile	8.4	7.1	9.7	18.1	16.4	19.8	32.1		30.1	34.2	10.3	9.1	11.6
*P*-value	<0.001			<0.001			<0.001				<0.001		
**Children ever born, n**													
0	17.2	13.9	20.5	15.9	12.9	18.9	19.7		15.9	23.6	6.1	3.1	9.1
1	18.9	16.9	20.9	16.0	13.6	18.4	21.6		18.9	24.2	5.4	4.3	6.6
2	18.7	16.2	21.1	15.3	13.6	17.0	20.9		19.1	22.8	5.1	4.1	6.1
3	18.5	14.1	22.9	13.0	11.4	14.6	20.5		12.3	28.8	3.9	3.2	4.7
≥4	19.2	15.6	22.9	9.9	8.0	11.9	13.2		10.6	15.8	3.5	0.9	6.1
*P*-value	<0.001			0.014			<0.001				<0.001		
**Media access**													
Low	27.8	25.4	30.3	11.3	10.1	12.4	10.4		9.1	11.8	1.4	1.0	1.8
Medium	15.9	14.7	17.2	16.9	15.6	18.2	20.8		19.6	21.9	4.7	4.0	5.3
High	10.1	8.5	11.7	17.6	15.4	19.7	30.6		28.3	32.9	9.6	8.1	11.2
*P*-value	<0.001			<0.001			<0.001				<0.001		
**Contraception use**													
Not using	21.8	20.0	23.6	13.6	12.2	15.0	17.8		16.1	19.4	4.3	3.6	5.1
Oral contraception	16.2	14.6	17.8	15.8	14.3	17.3	18.3		16.8	19.8	3.4	2.5	4.3
Others	19.8	18.2	21.4	15.8	14.3	17.3	18.5		16.9	20.2	4.1	3.3	3.5
*P*-value	<0.001			0.009			0.089				0.049		
**Household decision making power index**													
0 of 3 items	22.3	20.6	24.0	12.2	10.9	13.6	15.0		13.6	16.4	3.6	2.9	4.4
1 of 3 items	20.5	18.3	22.7	17.1	15.2	19.1	17.3		15.4	19.2	3.0	2.3	3.8
2 of 3 items	19.3	17.4	21.1	15.3	13.4	17.2	19.6		17.9	21.3	4.0	3.0	5.0
All 3 items	17.8	15.9	19.8	15.7	14.5	16.9	19.4		17.8	21.0	4.6	3.8	5.4
*P*-value	<0.001			<0.001			<0.001				0.001		
**Community wealth status**													
Low	27.0	25.0	29.1	11.2	10.2	12.2	10.3		8.9	11.7	1.1	0.7	1.5
Medium	20.0	18.6	21.3	16.2	14.6	17.7	17.1		15.8	18.4	3.1	2.6	3.7
High	11.3	10.1	12.6	17.7	16.4	19.1	27.5		26.0	29.0	8.0	6.9	9.2
*P*-value	<0.001			<0.001			<0.001				<0.001		
**Community illiteracy status**													
Low	14.3	12.8	15.8	17.0	15.7	18.3	24.6		23.0	26.2	6.0	4.9	7.1
Medium	19.0	17.2	20.9	15.8	14.2	17.3	17.4		15.8	19.0	4.2	3.3	5.0
High	25.0	23.1	26.9	12.3	11.2	13.4	13.0		11.2	14.7	2.1	1.5	2.8
*P*-value	<0.001			<0.001			<0.001				<0.001		
**Living environment**													
Rural	22.1	20.8	23.5	14.3	13.4	15.2	15.1		13.9	16.3	2.4	2.0	2.8
Urban	13.5	11.5	15.5	16.4	14.9	18.0	25.6		23.9	27.3	8.1	6.8	9.3
*P*-value	<0.001			0.030			<0.001				<0.001		
**Region**													
Barisal	21.8	17.7	26.0	13.3	11.8	14.9	16.3		13.3	19.2	4.2	0.6	7.8
Chittagong	16.4	14.4	18.3	14.8	13.0	16.6	20.7		18.3	23.0	5.1	3.9	6.3
Dhaka	19.1	16.4	21.7	15.9	14.3	17.5	19.3		16.5	22.1	4.2	3.2	5.3
Khulna	15.7	13.9	17.5	15.9	14.3	17.6	21.1		19.3	22.9	4.6	3.5	5.4
Rajshahi	21.7	19.2	24.2	15.0	11.8	14.9	17.5		15.4	19.6	3.7	2.8	4.5
Rangpur	21.2	17.9	24.6	13.7	11.0	16.3	13.4		11.1	15.7	2.6	1.6	3.5
Sylhet	30.7	27.2	34.2	11.1	9.6	12.6	11.2		9.6	12.8	2.7	1.8	3.5
*P*-value	<0.001			0.051			<0.001				0.171		

AARI and AARR values between the 2004 and 2014 BDHS are presented in Table 2. Between 2004 and 2014, as compared with women with no education (4.9%), there was a higher change in the annual rate of undernutrition among women with higher education (6.0%). Also, in comparison with higher-educated women, women with no education had higher AARI values for at-risk for overweightness (2.8% vs. 7.7%), overweightness (3.0% vs. 11.7%), and obesity (5.8% vs. 20.3%). According to household wealth index, the top quintile experienced a higher rate of decrease in underweightness (8.7% vs. 3.7%) than did the bottom quintile, while the women from the bottom quintile experienced a higher rate of increase in being at-risk for overweightness (10.1% vs. 2.2%), overweightness, (14.7% vs. 5.7%), and obesity (15.4% vs. 9.6%) than did the top-quintile women. However, changes in the four weight categories among women between 2004 and 2014 also showed similar patterns regarding individual education and household wealth when they were categorized according to community wealth and community illiteracy status.

**Table 2:** Annual average rate of reduction or increase in the prevalence of underweight, at risk for overweight, overweight and obese among Bangladeshi women aged 15–49 by individual socio-demographic and community-level variables, 2004–2014.

Variables	Sample Size		BMI (kg/m^2^)								Percent AARR/AARI			
	2004 (n = 10431)	2014 (n = 16478)	<18.5		23–24.9		25–29.9		≥30		<18.5^a^	23–24.9^b^	25–29.9^b^	≥30^b^
			2004 n (%)	2014 n (%)	2004 n (%)	2014 n (%)	2004 n (%)	2014 n (%)	2004 n (%)	2014 n (%)				
**Age**														
15–19	1303	1619	514 (39.7)	527 (31.0)	49 (4.0)	156 (9.7)	20 (1.5)	99 (5.8)	4 (0.3)	18 (1.3)	2.46	9.17	14.35	16.50
20–24	1923	2774	682 (36.3)	635 (22.8)	122 (5.9)	351 (13.4)	116 (5.1)	399 (14.1)	16 (0.6)	60 (2.1)	4.54	8.53	10.74	12.98
25–29	1819	3112	552 (31.0)	497 (16.8)	180 (9.3)	502 (16.2)	155 (7.1)	635 (20.5)	29 (1.4)	10.8 (3.2)	5.95	5.68	11.23	8.50
30–34	1671	2899	497 (31.5)	397 (13.5)	175 (9.8)	490 (17.0)	177 (9.2)	719 (24.8)	41 (2.1)	167 (5.9)	8.11	5.71	10.46	11.19
35–39	1405	2266	407 (30.2)	332 (13.6)	153 (9.9)	389 (17.6)	186 (11.8)	547 (23.5)	34 (1.5)	150 (6.4)	7.66	5.96	7.10	15.46
40–44	1140	2065	389 (34.4)	356 (17.8)	122 (10.3)	379 (17.2)	138 (10.4)	491 (21.9)	35 (2.4)	131 (6.1)	6.37	5.30	7.74	9.69
45–49	1048	1743	410 (40.1)	330 (19.9)	96 (9.7)	245 (14.8)	119 (9.5)	397 (21.1)	25 (1.9)	108 (5.7)	6.77	4.34	8.36	11.85
**Education**														
None	4393	4255	1659 (40.1)	983 (24.2)	282 (6.7)	535 (14.1)	197 (4.4)	566 (13.2)	17 (0.4)	106 (2.6)	4.94	7.72	11.72	20.33
Primary	2988	4810	1066 (35.0)	991 (20.7)	246 (8.0)	714 (14.5)	221 (6.6)	834 (17.1)	43 (1.3)	178 (3.7)	5.11	6.11	9.89	10.96
Secondary	2415	6051	685 (27.3)	965 (5.3)	274 (10.0)	962 (16.3)	335 (10.8)	1389 (22.8)	89 (2.6)	332 (5.4)	5.62	5.04	7.72	7.66
Higher	513	1361	87 (16.3)	35 (8.8)	98 (14.2)	301 (18.7)	158 (23.8)	498 (32.0)	35 (4.4)	126 (7.8)	5.97	2.82	3.01	5.82
**Current marital status**														
Married	9481	15488	3132 (33.6)	2812 (18.1)	843 (8.4)	2381 (15.6)	847 (7.6)	3138 (19.7)	172 (1.4)	699 (4.4)	6.01	6.42	10.00	12.16
Widowed	484	615	212 (46.2)	153 (27.8)	34 (7.2)	88 (13.9)	43 (7.4)	107 (15.4)	7 (0.8)	27 (4.0)	4.95	6.80	7.57	17.15
Divorced/Separated	244	374	1153 (39.4)	109 (25.4)	23 (5.8)	43 (10.3)	21 (4.8)	42 (10.8)	5 (1.8)	16 (3.9)	4.29	5.93	8.45	7.95
**Religion**														
Non-Muslim	1034	1634	386 (32.8)	296 (16.3)	105 (8.9)	251 (15.1)	93 (7.4)	303 (20.0)	16 (1.4)	72 (5.4)	6.78	5.36	10.46	14.39
Muslim	9269	14843	3108 (34.6)	2778 (18.9)	795 (8.2)	2260 (15.4)	818 (7.5)	2984 (19.3)	168 (1.4)	670 (4.3)	5.87	6.60	9.90	11.96
**Employment status**														
Homemaker	7896	10416	2637 (33.6)	2005 (18.4)	707 (8.4)	1630 (15.5)	743 (8.0)	2233 (20.8)	165 (1.6)	564 (5.3)	5.82	6.37	10.06	12.56
Agricultural	779	2783	244 (37.9)	515 (21.1)	47 (6.7)	365 (15.0)	26 (3.8)	379 (13.6)	6 (0.7)	54 (2.1)	5.70	8.34	13.57	11.46
Non-manual	246	1338	84 (34.2)	179 (13.3)	24 (9.5)	227 (16.2)	35 (11.3)	308 (21.5)	7 (1.5)	54 (3.5)	9.02	5.51	6.69	9.16
Manual	1383	1908	529 (37.3)	372 (20.0)	121 (8.1)	281 (14.7)	106 (6.3)	358 (18.6)	6 (0.4)	70 (3.4)	6.03	6.13	11.51	24.14
**Household wealth index**														
Bottom quintile	2043	3057	888 (47.2)	946 (32.3)	64 (3.6)	302 (9.4)	33 (1.9)	246 (7.4)	4 (0.2)	25 (1.0)	3.73	10.05	14.67	15.36
Second quintile	2052	3116	782 (40.7)	775 (25.0)	80 (4.5)	413 (13.9)	46 (2.5)	365 (12.2)	6 (0.4)	36 (1.2)	4.77	11.95	17.22	12.30
Third quintile	2024	3299	717 (35.8)	645 (19.1)	139 (7.7)	510 (14.6)	88 (4.5)	573 (17.4)	8 (0.4)	88 (2.7)	6.09	6.56	14.42	20.07
Fourth quintile	2105	3496	665 (31.3)	453 (12.3)	218 (10.8)	650 (20.2)	169 (8.3)	808 (22.3)	23 (1.2)	176 (4.6)	8.93	6.51	10.40	14.20
Top quintile	2085	3509	445 (17.3)	255 (7.0)	399 (14.5)	637 (18.0)	575 (20.2)	1295 (35.2)	143 (4.6)	417 (11.5)	8.68	2.22	5.71	9.60
**Children ever born, n**														
0	884	1241	331 (31.0)	304 (21.7)	65 (6.3)	181 (13.8)	64 (5.7)	173 (13.1)	7 (0.8)	36 (3.6)	3.49	8.20	8.77	15.77
1	1747	3551	615 (35.6)	791 (22.0)	140 (6.8)	513 (14.4)	141 (6.5)	629 (16.8)	28 (1.2)	139 (3.2)	4.70	7.70	10.05	10.31
2	2132	4475	627 (30.3)	612 (14.3)	241 (10.5)	756 (17.0)	223 (8.8)	1088 (23.4)	54 (1.8)	237 (5.4)	7.26	4.95	10.29	11.35
3	1834	3276	532 (30.9)	519 (16.0)	159 (8.7)	521 (16.7)	185 (8.6)	690 (21.0)	40 (1.7)	185 (5.5)	6.36	6.68	9.35	12.31
≥4	3712	3934	1392 (38.9)	848 (21.8)	295 (7.9)	541 (14.0)	298 (7.2)	707 (17.7)	55 (1.2)	145 (3.6)	5.63	5.94	9.38	11.80
**Media access**														
Low	4045	6155	1690 (43.6)	1610 (26.8)	205 (5.2)	730 (11.8)	121 (3.2)	698 (11.5)	20 (0.5)	95 (1.7)	4.75	8.51	13.54	13.68
Medium	3129	8093	1001 (32.6)	1232 (15.0)	290 (8.8)	1361 (17.5)	258 (7.2)	1828 (22.0)	51 (1.4)	425 (5.0)	7.50	7.07	11.81	13.62
High	332	2199	805 (24.5)	222 (9.2)	405 (11.5)	417 (17.8)	529 (13.3)	754 (31.9)	113 (2.6)	221 (9.8)	9.35	4.47	9.18	14.38
**Current contraception method**														
Not using	4213	6027	1680 (39.2)	1306 (21.7)	293 (6.5)	856 (13.6)	325 (6.6)	1170 (18.7)	71 (1.3)	299 (4.8)	5.77	7.67	11.02	13.77
Oral contraception	2729	4523	694 (27.4)	741 (15.7)	267 (9.3)	715 (16.2)	226 (7.3)	853 (18.6)	30 (0.8)	147 (3.5)	5.43	5.72	9.76	16.31
Others	3367	5927	1123 (34.0)	1027 (17.8)	340 (9.6)	941 (16.6)	360 (8.8)	1264 (20.6)	83 (2.0)	296 (4.7)	6.27	5.69	8.88	8.98
**Household decision-making power index**														
0 of 3 items	3502	4180	1342 (38.7)	1013 (22.4)	234 (6.4)	559 (12.3)	181 (4.2)	642 (15.2)	28 (0.5)	161 (3.8)	5.35	6.71	13.76	21.45
1 of 3 items	1872	2331	607 (33.2)	502 (20.0)	174 (9.2)	385 (17.5)	158 (7.5)	446 (17.6)	25 (1.2)	85 (3.1)	4.95	6.67	8.89	9.61
2 of 3 items	1897	2493	592 (32.4)	444 (18.1)	193 (9.0)	391 (15.8)	200 (9.3)	548 (21.4)	37 (1.6)	116 (4.3)	5.63	5.72	8.71	10.56
3 of 3 items	3038	7473	956 (31.3)	444 (16.3)	299 (9.3)	1177 (16.4)	372 (10.3)	1651 (21.6)	94 (2.4)	380 (5.2)	6.32	5.87	7.68	8.18
**Community wealth status**														
Low	3954	6182	1497 (42.6)	1477 (26.3)	157 (4.8)	645 (11.8)	93 (2.6)	605 (10.9)	11 (0.3)	75 (1.2)	4.69	9.46	15.26	16.37
Medium	3686	5276	1191 (32.9)	1023 (18.9)	318 (9.5)	876 (16.7)	237 (7.0)	1027 (18.3)	33 (1.1)	187 (3.5)	5.42	5.82	10.12	12.14
High	2669	5019	809 (23.7)	574 (10.0)	425 (11.9)	991 (18.2)	581 (15.9)	1655 (29.6)	140 (3.5)	480 (8.7)	8.25	4.30	6.45	9.43
**Community illiteracy status**														
Low	3954	6182	1459 (40.8)	1406 (25.3)	194 (5.7)	706 (12.9)	146 (4.1)	733 (12.6)	16 (0.4)	127 (2.0)	4.66	8.50	11.86	18.92
Medium	3686	5276	1181 (33.9)	1052 (17.6)	296 (8.7)	814 (15.2)	231 (6.1)	1042 (19.5)	35 (1.0)	217 (4.3)	6.34	5.79	12.27	15.14
High	2669	5019	857 (25.8)	616 (11.5)	410 (11.3)	992 (18.8)	534 (14.4)	1512 (27.6)	133 (3.4)	398 (7.5)	7.79	5.18	6.71	8.22
**Living environment**														
Rural	7968	11792	2553 (37.1)	2340 (21.2)	492 (7.5)	1555 (14.9)	346 (5.2)	1701 (16.1)	42 (0.7)	279 (2.6)	5.46	7.06	12.08	14.70
Urban	2341	4685	898 (24.9)	734 (12.2)	405 (11.0)	957 (16.8)	565 (15.9)	1586 (27.6)	142 (3.9)	463 (8.8)	6.87	4.31	5.65	8.47

AARR: Annual average rate of reduction; AARI: Annual average rate of increase

^a^AARR in the prevalence of underweight

^b^AARI in the prevalence of risk for overweight, overweight, and obese

Table 3 presents the adjusted odds ratios (AORs) and the 95% CIs for the risk of being underweight, at-risk for overweight, overweight, and obese for the exposure variable as well as the exposures and covariates, using the normal BMI category as the reference. At the individual level, women with secondary education (as compared with no education) were significantly and negatively associated with underweightness (AOR: 0.82, 95% CI: 0.70–0.95). However, in comparison with women with no education, higher-educated women had a greater odds of being at-risk for overweightness (AOR: 1.58, 95% CI: 1.23–2.04), overweight (AOR: 1.74, 95% CI: 1.36–2.23), and obese (AOR: 1.62; 95% CI: 1.08–2.44). Women in the top quintile of the household wealth (in comparison with the bottom quintile) were significantly less likely to be underweight (AOR: 0.50, 95% CI: 0.39–0.65) and more likely to be at-risk for overweightness, overweight, and obese. Women with higher media access had 1.33, 1.60, and 2.00 times higher odds of being at-risk for overweightness, overweight, and obese, respectively. At the community level, women in the wealthiest community were significantly and positively associated with being at-risk for overweightness (AOR: 1.53, 95% CI: 1.23–1.91), overweightness (AOR: 1.60, 95% CI: 1.27–2.00), and obese (AOR: 2.12, 95% CI: 1.42–3.18).

**Table 3:** Adjusted^a^ OR and 95% CI from a multilevel multinomial logistic regression predicting underweight, at risk for overweight, overweight and obese for ever-married Bangladeshi women aged 15–49 years from the BDHS 2014^b^.

Variables	Underweight BMI < 18.5 (n = 3069)		At risk for overweight BMI ≥ 23.0 & < 25.0 (n = 2538)		Overweight BMI ≥ 25.0 & < 30.0 (n = 3193)		Obese BMI ≥ 30.0 (n = 722)	
	UOR (95% CI)	AOR (95% CI)	UOR (95% CI)	AOR (95% CI)	UOR (95% CI)	AOR (95% CI)	UOR (95% CI)	AOR (95% CI)
**Education**								
None	1	1	1	1	1	1	1	1
Primary	0.88 (0.76–1.02)	0.90 (0.78–1.04	1.05 (0.86–1.28)	1.19 (1.03–1.38)	1.32 (1.09–1.60)	1.30 (1.11–153)	1.43 (1.07–1.90)	1.47 (1.14–1.900
Secondary	0.67 (0.55–0.81)	0.82 (0.70–0.95)	1.22 (0.98–1.52)	1.33 (1.12–1.57)	1.82 (1.56–2.15)	1.64 (1.38–1.95)	2.20 (1.65–2.94)	1.79 (1.35–2.37)
Higher	0.44 (0.32–0.59)	0.78 (0.59–1.04	1.59 (1.22–2.07)	1.58 (1.23–2.04)	2.90 (2.33–3.62)	1.74 (1.36–2.23)	3.58 (2.55–5.03)	1.62 (1.08–2.44)
**Household wealth**								
Bottom quintile	1	1	1	1	1	1	1	1
Second quintile	0.79 (0.68–0.92)	0.84 (0.73–0.98)	1.52 (1.20–1.91)	1.15 (0.95–1.39)	1.69 (1.34–2.13)	1.19 (0.98–1.46)	1.26 (0.74–2.13)	1.12 (0.68–1.83)
Third quintile	0.61 (0.53–0.72)	0.70 (0.60–0.94)	1.61 (1.33–1.96)	1.19 (0.98–1.45)	2.45 (1.96–3.06)	1.48 (1.22–1.81)	2.95 (1.69–5.12)	1.94 (1.20–3.16)
Fourth quintile	0.45 (0.37–0.54)	0.56 (0.46–0.68)	2.54 (1.86–3.84)	1.47 (1.19–1.81)	3.56 (2.81–4.51)	1.91 (1.55–2.37)	5.68 (3.46–9.32)	3.26 (2.02–5.24)
Top quintile	0.37 (0.31–0.46)	0.50 (0.39–0.65)	3.28 (2.62–4.10)	1.79 (1.38–2.34)	8.15 (6.44–10.30)	3.51 (2.70–4.57)	20.41 (12.39–33.64)	7.90 (4.70–13.28)
**Media access**								
Low	1	1	1	1	1	1	1	1
Medium	0.61 (0.52–0.73)	0.95 (0.84–1.08)	1.62 (1.40–1.87)	1.23 (1.06–1.42)	2.10 (1.81–2.44)	1.31 (1.13–1.52)	3.14 (2.26–4.36)	1.44 (1.11–1.87)
High	0.46 (0.35–0.60)	0.96 (0.77–1.19)	2.01 (1.62–2.50)	1.33 (1.08–1.66)	3.73 (3.04–4.56)	1.60 (1.30–1.97)	7.56 (5.13–11.13)	2.00 (1.41–2.85)
**Community wealth status**								
Low	1	1	1	1	1	1	1	1
Medium	0.82 (0.73–0.94)	1.03 (0.89–1.20)	1.62 (1.39–1.90)	1.26 (1.08–1.47)	1.93 (11.62–2.30)	1.25 (1.06–1.48)	3.22 (2.16–4.82)	1.43 (1.02–2.00)
High	0.58 (0.49–0.66)	0.99 (0.79–1.25)	2.33 (2.01–2.71)	1.53 (1.23–1.91)	4.11 (3.44–4.91)	1.60 (1.27–2.00)	10.63 (7.17–15.74)	2.12 (1.42–3.18)
**Community illiteracy status**								
Low		1		1		1		1
Medium		1.05 (0.89–1.23)		0.99 (0.86–1.14)		0.95 (0.82–1.10)		1.23 (0.99–1.53)
High		1.07 (0.90–1.28		1.02 (0.88–1.21)		1.08 (0.92–1.26)		1.31 (1.00–1.72

Note: UOR, unadjusted odds ratio; AOR, adjusted odds ratio; CI, Confidence interval.

^a^Adjusted for age, marital status, religion, employment status, children ever born, household decision making power, living environment and region.

^b^Using BMI (in kg/m^2^) between 18.5 and 23.0 as the reference, n = 6956

Table 4 presents the results obtained in the quantile regression analysis. It illustrates how the effects of individual and community-level variables on women’s BMI vary among quantiles. Of note, the effect of higher education was significantly positive in all quantiles except in the 90^th^ quantile. Thus, women who were more educated achieved increases of 1.109, 1.110, 0.890, 0.720, and 0.262 in BMI in the 10^th^, 25^th^, 50^th^ 75^th^, and 90^th^quintile, respectively. However, the effect of household wealth increased for individuals across all quantiles. The effect of the top-quintile household’s (versus the bottom quintile) wealth on BMI was monotonically positive, with magnitudes that become increasingly stronger upon moving from the lowest 10^th^ quantile to the highest 90^th^ quantile. A similar increment pattern was also observed among women with high media access versus in their counterparts with low media access. The significant positive effects of the household decision-making index on BMI of the women were observed in all quantiles. Women who participated in all three items of household decision-making were associated with increases of 0.227, 0.273, 0.293, 0.387, and 0.486 units in BMI for the 10^th^, 25^th^, 50^th^ 75^th^, and 90^th^ quantiles, respectively. Except in the 10^th^ quantile, high community wealth was also positively associated with women’s BMI in all quantiles. Living in an illiterate community was found to have a significantly negative effect on BMI of women in the 25^th^ quantile only. Living in a high illiterate community was associated with a decrease of 0.192 units in BMI for those in the 25^th^ quantile.

**Table 4:** Factors associated with quantiles of BMI among women aged 15–49 in Bangladesh.

Variables	Q 10				Q 25				Q 50				Q 75				Q 90			
	Coef	p	95% CI		Coef	p	95% CI		Coef	p	95% CI		Coef	p	95% CI		Coef	p	95% CI	
			low	high			low	high			low	high			low	high			low	high
**Intercept**	15.140	<0.001	14.631	15.631	16.108	<0.001	15.647	16.569	17.241	<0.001	16.737	17.744	18.862	<0.001	18.240	19.485	21.328	<0.001	20.490	22.167
**Age**																				
15–19	1				1				1				1				1			
20–24	0.397	0.003	0.136	0.658	0.593	<0.001	0.359	0.828	1.092	<0.001	0.828	1.358	1.442	<0.001	1.101	1.783	1.625	<0.001	1.146	2.106
25–29	1.052	<0.001	0.782	1.321	1.539	<0.001	1.271	1.808	2.302	<0.001	2.002	2.602	2.688	<0.001	2.320	3.056	2.745	<0.001	2.228	3.262
30–34	1.608	<0.001	1.280	1.935	2.256	<0.001	1.958	1.555	3.213	<0.001	2.879	3.548	3.656	<0.001	3.256	4.056	3.789	<0.001	3.180	4.397
35–39	1.787	<0.001	1.424	1.151	2.605	<0.001	2.281	2.928	3.502	<0.001	3.156	3.849	3.959	<0.001	3.523	4.394	4.019	<0.001	3.378	4.660
40–44	1.634	<0.001	1.159	1.968	2.627	<0.001	2.284	2.970	3.442	<0.001	3.045	3.799	3.862	<0.001	3.409	4.315	4.077	<0.001	3.414	4.740
45–49	1.564	<0.001	1.159	1.968	2.361	<0.001	1.990	2.73	3.263	<0.001	2.857	3.669	3.877	<0.001	3.412	4.341	3.684	<0.001	2.999	4.370
**Marital status**																				
Married	1				1				1				1				1			
Widowed	-0.381	0.054	-0.768	0.006	-0.701	0.004	-1.172	-0.230	-0.566	0.012	-1.001	-0.123	-0.459	0.124	-1.044	0.126	-0.373	0.345	-1.146	0.401
Divorced/Separated	-0.362	0.104	-0.799	0.074	-0.602	0.006	-1.032	-0.172	-1.087	<0.001	-1.616	-0.558	-0.988	0.001	-1.586	-0.389	-0.836	0.099	-1.829	0.156
**Religion**																				
Non-Muslins	1				1															
Muslims	-0.088	0.413	-0.300	0.123	-0.045	0.680	-0.257	0.167	0.096	0.380	-0.118	0.311	0.174	0.239	-0.116	0.464	0.445	0.040	0.020	0.870
**Education**																				
None	1				1				1				1				1			
Primary	0.333	0.002	0.127	0.538	0.417	<0.001	0.222	0.612	0.511	<0.001	0.312	0.709	0.615	<0.001	0.386	0.845	0.507	0.006	0.146	0.867
Secondary	0.762	<0.001	0.508	1.017	0.829	<0.001	0.611	1.048	0.860	<0.001	0.628	1.092	0.978	<0.001	0.708	1.247	0.901	<0.001	0.501	1.300
Higher	1.109	<0.001	0.731	1.487	1.110	<0.001	0.781	1.439	0.890	<0.001	0.532	1.248	0.720	0.001	0.311	1.130	0.262	0.408	-0.359	0.883
**Employment status**																				
Homemaker	1				1				1				1				1			
Agricultural	0.096	0.321	-0.285	0.093	-0.265	0.008	-0.459	-0.070	-0.284	0.003	-0.474	-0.095	-0.303	0.011	-0.538	-0.069	-0.490	0.004	-0.824	-0.156
Non-manual	0.097	0.521	-0.199	0.292	-0.129	0.248	-0.347	0.090	-0.329	0.013	-0.589	0.069	-0.394	0.012	-0.704	-0.085	-0.570	0.021	-1.055	-0.085
Manual	-0.341	0.005	-0.579	-0.104	-0.369	<0.001	-0.573	0.164	-0.486	<0.001	-0.676	-0.269	-0.517	<0.001	-0.785	-0.249	-0.533	0.011	-0.942	-0.123
**Household wealth index**																				
Bottom quintile	1				1				1				1				1			
Second quintile	0.075	0.470	-0.128	0.277	0.236	0.016	0.044	0.427	0.275	<0.001	0.079	0.472	0.342	0.017	0.062	0.622	0.486	0.012	0.105	0.868
Third quintile	0.403	0.001	0.174	0.632	0.528	<0.001	0.320	0.737	0.698	<0.001	0.480	0.916	0.842	<0.001	0.532	1.152	1.223	<0.001	0.769	1.678
Fourth quintile	0.805	<0.001	0.530	1.080	1.111	<0.001	0.866	1.355	1.394	<0.001	1.142	1.646	1.592	<0.001	1.244	1.941	1.954	<0.001	1.462	2.447
Top quintile	1.757	<0.001	1.428	2.087	2.186	<0.001	1.882	2.489	2.743	<0.001	2.395	3.091	2.936	<0.001	2.501	3.371	3.435	<0.001	2.891	3.979
**Children ever born, n**																				
0	1				1				1				1				1			
1	-0.053	0.743	-0.369	0.263	-0.091	0.490	-0.348	0.167	-0.030	0.859	-0.363	0.303	-0.065	0.744	-0.454	0.324	-0.213	0.433	-0.747	0.320
2	0.426	0.015	0.082	0.771	0.411	0.010	0.098	0.724	0.222	0.229	-0.140	0.584	0.045	0.836	-0.388	0.479	-0.007	0.982	-0.643	0.628
3	0.113	0.574	-0.280	0.505	0.031	0.860	-0.311	0.372	-0.263	0.199	-0.664	0.138	-0.383	0.131	-0.879	0.114	-0.389	0.266	-1.074	0.296
≥4	-0.273	0.197	-0.688	0.142	-0.473	0.013	-0.845	-0.102	-0.642	0.002	-1.054	-0.229	-0.786	0.002	-0.126	-0.285	-0.766	0.035	-1.479	-0.054
**Media access**																				
Low	1				1				1				1				1			
Medium	0.159	0.082	-0.215	0.608	0.315	<0.001	0.161	0.468	0.392	<0.001	0.234	0.550	0.493	<0.001	0.265	0.721	0.442	0.006	0.125	0.760
High	0.396	0.006	0.069	0.440	0.566	<0.001	0.296	0.835	0.640	<0.001	0.326	0.956	0.842	<0.001	0.477	1.208	1.262	<0.001	0.787	1.736
**Current contraception method**																				
Not using	1				1				1				1				1			
Oral contraception	0.412	<0.001	0.215	0.608	0.358	<0.001	0.178	0.539	0.147	0.119	-0.038	0.331	-0.065	0.591	-0.302	0.172	-0.544	0.001	-0.881	-0.208
Others	0.255	0.007	0.069	0.440	0.195	0.021	0.029	0.361	0.070	0.444	-0.110	0.250	-0.024	0.839	-0.839	0.209	-0.350	0.050	-0.700	-0.001
**Household decision-making power**																				
0 of 3 items	1				1				1				1				1			
1 of 3 items	0.015	0.897	-0.217	0.247	0.009	0.931	-0.200	0.219	0.032	0.773	-0.188	0.252	0.111	0.451	-0.178	0.400	0.130	0.520	-0.266	0.525
2 of 3 items	-0.013	0.924	-0.269	0.244	0.157	0.174	-0.069	0.382	0.084	0.474	-0.146	0.313	0.210	0.146	-0.073	0.493	0.350	0.121	-0.093	0.793
All 3 items	0.227	0.022	0.032	0.422	0.273	0.002	0.090	0.448	0.293	0.001	0.117	0.469	0.387	0.002	0.143	0.631	0.486	0.003	0.60	0.813
**Community wealth status**																				
Low	1				1				1				1				1			
Medium	0.125	0.232	-0.080	0.329	0.189	0.028	0.020	0.359	0.277	0.003	0.096	0.458	0.362	0.006	0.106	0.618	0.274	0.117	-0.069	0.617
High	0.199	0.224	-0.121	0.519	0.053	<0.001	0.274	0.797	0.820	<0.001	0.544	1.096	0.829	<0.001	0.460	1.199	0.658	0.014	0.135	1.181
**Community illiteracy status**																				
Low	1				1				1				1				1			
Medium	-0.104	0.307	-0.304	0.096	-0.249	0.004	-0.421	-0.077	-0.095	0.322	-0.282	0.093	0.008	0.947	-0.226	0.242	0.131	0.410	-0.181	0.444
High	0.010	0.927	-0.212	0.232	-0.192	0.045	-0.379	-0.004	-0.013	0.901	-0.214	0.188	0.162	0.194	-0.082	0.406	0.304	0.29	-0.088	0.697
**Living environment**																				
Rural	1				1				1				1				1			
Urban	0.220	0.032	0.019	0.422	0.196	0.039	0.010	0.382	0.219	0.025	0.027	0.411	0.528	<0.001	0.310	0.746	0.733	<0.001	0.382	1.083
**Region**																				
Barisal	1				1				1				1				1			
Chittagong	0.075	0.603	-0.209	0.359	0.035	0.794	-0.226	0.295	0.298	0.025	0.037	0.558	0.182	0.327	-0.182	0.546	-0.105	0.698	-0.634	0.425
Dhaka	0.022	0.879	-0.259	0.302	-0.057	0.672	-0.323	0.208	-0.252	0.055	-0.511	0.006	-0.144	0.420	-0.495	0.207	-0.576	0.015	-1.044	-0.111
Khulna	0.383	0.009	0.097	0.671	0.328	0.013	0.069	0.586	0.489	<0.001	0.232	0.747	0.644	0.001	0.247	1.041	0.445	0.089	-0.068	0.958
Rajshahi	0.110	0.465	-0.184	0.404	0.024	0.864	-0.252	0.300	0.160	0.208	-0.089	0.410	0.224	0.264	-0.169	0.618	-0.004	0.988	-0.502	0.494
Rangpur	0.070	0.652	-0.232	0.372	0.155	0.234	-0.100	0.410	0.113	0.388	-0.144	0.369	0.069	0.713	-0.298	0.436	-0.174	0.488	-0.667	0.319
Sylhet	-0.852	<0.001	-1.151	-0.554	-0.791	<0.001	-1.067	-0.516	-0.880	<0.001	-1.166	-0.593	-0.838	<0.001	-1.219	-0.456	-0.352	<0.001	-1.889	-0.817
Pseudo R^2^	0.076				0.110				0.139				0.144				0.138			

## Discussion

Drawing on a nationally representative dataset, this study comprehensively examined the prevalence of and individual socioeconomic and community-level factors associated with the double burden of malnutrition in Bangladesh. We found that, in 2014, the age-adjusted prevalence values of underweightness, at-risk for underweightness, overweightness, and obesity among women were19.7%, 14.9%, 18.1%, and 4.0%, respectively, which means that more than half of Bangladeshi women face a potential nutritional problem. An earlier study in Bangladesh using 2011 BDHS data and similar BMI cut-offs showed that the prevalence of underweight, at risk for underweight, overweight and obese among women was 24.1%, 12.8%, 13.5% and 2.9.0%, respectively [14], indicating that prevalence of underweight among women is decreasing while overweight and obese are increasing steadily. The prevalence of underweightness, and overweightness or obesity found in this study were higher than China and Myanmar [47, 48]. These data also suggest the coexistence of underweight and overweight in Bangladesh and that the prevalence of overweight (at-risk for overweight, overweight and obese) surpasses the prevalence of underweight. This double burden of malnutrition is shared roughly by 1:2 ratios of undernutrition and overnutrition problems. This co-existence of the under- and overnutrition among Bangladeshi women is likely to reflect the differential distribution of resources at the individual level that means some women do not have enough resources to get their daily caloric requirements whereas others, by their enough purchasing capacity, cannot only meet but also exceed their caloric requirements.

The findings of this study suggest that higher AARR of underweight is likely to occur among women who are highly educated, live in wealthy household and wealthier communities. On the contrary, AARI of at-risk for overweightness, overweightness and obese are greater among women with no education, poor households and living in poor communities. Higher increase in the rate of at-risk for overweightness, overweightness and obese among poor and/or uneducated women in Bangladesh suggests disparities in the burden of overweight. The annual average change in the prevalence of at-risk for overweightness, overweightness and obesity was found higher among women in the poor communities. This may be due to the readily available amenities such as television, vehicle access, sedentary life styles and restaurants that have increased in poor communities which might be responsible for reducing physical activities and unhealthy eating. Initially, the emergence of overweightness and obesity were associated with higher socio-economic group of the populations in developing countries [49]. However, recent trends document a shift in the prevalence of overweightness and obesity from higher to lower socioeconomic groups [49]. For example, increasing rate of overweightness among lower compared to higher socioeconomic groups have been documented in Brazil [50] and in urban areas of sub-Saharan Africa [51]. Multi-country studies [52, 53], examined the overweight prevalence growth rates over time and found that overweight has increased more for the lowest wealth or education groups than the highest groups in as substantial portion of the sample countries. Underlying cause of this phenomenon of experiencing higher growth rate of overweightness prevalence among lowest wealth or education groups might be explained by lower wealth or education groups are experiencing the same environmental changes, such as accessibility of energy-dense foods, labour-saving devices and sedentary occupation, that high wealth or education groups experienced earlier [53]. On the other hand, higher wealth or education groups might have increased medical knowledge and are concern about the overweight/obesity and/or stigma around larger body sizes, which may lead them to respond to the changing conditions [53, 54], and resulted in slower growth rate of overweight prevalence among these populations.

The present analysis found significant nutritional inequalities among individual-level (household wealth, education and media access) and community-level wealth status of the women. Household wealth inequality is observed in this study for the risk of being underweight as well as each of the overnutrition categories (i.e., at-risk for overweightness, overweightness, and obesity). Consistent with earlier studies [14, 16, 20, 23, 24, 55], the present investigation found that women in the low-household-wealth quintile were more likely to be underweight, while those in the top-household-wealth quintile experience the greatest risk for being at-risk for overweightness, overweightness, and obesity. Therefore, the findings of this study suggest that, in Bangladesh, the double burden of underweightness and overweightness are not concentrated in the same population groups but rather overnutrition is a disease that primarily afflicts the affluent but is increasing rapidly among the poor.

This study found a clear distribution of malnutrition by individual education and household wealth among ever-married Bangladeshi women aged 15 to 49 years. Women with no education were more likely to be underweight, whereas women with higher education were more likely to be at-risk for overweightness, overweight, and obese. This finding is consistent with those of other studies from around the world [16, 19, 21, 23, 24, 56]. This indicates that, though women with higher education might have greater knowledge of how to overcome undernutrition, due to their higher income and greater independence, they may live a life with less physical activities and higher access to energy-dense foods, which are considered to be the cause of overweightness and obesity [57].

Our study additionally found that, although access to media was not significantly associated with undernutrition, it was positively and significantly associated with being at-risk for overweightness, overweightness, and obesity. This finding corroborates with results from both developing and developed countries, where television-watching was shown to be associated with being overweight and obese [58, 59]. Possible explanations include that television commercials may promote unhealthy eating habits and that watching television promotes physical inactivity.

Community-level variables (community wealth and community illiteracy status) presented mixed results in this study. While community illiteracy status was not significantly associated with women’s malnutrition, community wealth had a significant impact on women being underweight, at-risk for overweightness, overweight, and obese. Similar to in an earlier study in Bangladesh [24], we found that high wealth communities were less likely to have underweight women. On the contrary, community wealth was associated with overweight categories in a graded manner. High wealth communities were more likely to include women who were at-risk for overweightness, overweight, and obese after adjusting for other covariates. This finding indicates that, in Bangladesh, the burden of overweightness primarily exists in affluent areas. The wealthiest communities were located in urban areas throughout the country, suggesting that overnutrition may be occurring more rapidly in economically developed areas where energy-dense foods and motorized transportation may be more accessible [60]. Overweightness among the wealthiest communities may also be explained by their access to surplus/excess food and a lower level of engagement in manual labour-intensive work [61]. In addition, in some parts of the country, a larger female body size might be considered as a symbol of maternity, nurturance, and affluence; therefore, women in such an area might prefer a larger body size [62, 63]. On the other hand, researchers also documented that gender discrimination in intra household food allocation among women is common in some poor societies, which might pose a greater risk of malnutrition to women [64].

Our study has several strengths and limitations that should be mentioned. The main strength is that it used data from national representative samples covering both rural and urban areas in Bangladesh. For both surveys, eligible women response rates were extremely high (98% in 2014 and 98.6% in 2004). Additionally, anthropometric measurements (height and weight) were collected by trained interviewers according to the internationally recommended standard protocol [65], which made it possible to compute accurate BMI values for individuals. However, due to the cross-sectional nature of the data, which did not permit us to incorporate the temporal dimension into the analysis, this study only shows the socioeconomic and community-level inequalities of the double burden of malnutrition among women in Bangladesh in 2014. Different subsets of the population may be affected by the nutrition transition in different ways, even though the study findings are related to nationally representative ever-married women aged 15 to 49 years and did not include older women and women younger than15 years; therefore, extrapolation of these findings to the general female population should be done with caution. However, the association between individual socioeconomic community-level variables and nutritional status among women and men showed similar patterns in earlier studies in Bangladesh and India [24, 36, 66], which strengthen the relevance of this study’s findings. However, this factor may limit the ability of this study to estimate the true burden of chronic diseases associated with undernutrition and overnutrition. In predicting obesity-related metabolic risk at the population level, other measures of body fat (e.g. waist circumference, body fat mass percentage) have little advantage over BMI [67] and, therefore, the use of BMI was appropriate in this study. Moreover, in areas where a substantial proportion of the population remains undernourished, BMI is the most commonly available measure for studying weight status. Finally, lower BMI values (<18.5 kg/m^2^) were used to define undernutriton, but, due to a lack of available data in the BDHSs, we were unable to examine other forms of undernutrition, such as micronutrient deficiency, and were also unable to adjust for food intake patterns that might have contributed to residual confounding of the study.

Based on the findings of this study, policy implication relating to the following points for dealing with the double burden of malnutrition could be suggested. Interventions should be taken to targeted communities to raise the overall level of socioeconomic status of the populations through education and better employment opportunities that would help them to increase purchasing power, and, in turn, enable them to afford enough food to fulfil their needs. However, it is documented that the risk of being overweight increases among the wealthiest households and communities, so a broad public educational campaign that promotes behavioural changes specifically in the spheres of physical activity and dietary patterns is needed. Since higher education is not always associated with better nutritional status, as suggested in this study, there is perhaps a need for better nutritional education in the academic curricula, which will not only promote behaviour changes but also help to form a healthier body image in the community. To educate people about healthy eating choices, a wide-ranging specific mass media campaign along with local level support is necessary. While evidence supports that mass-media health campaigns can have a significant impact on awareness, attitudes, knowledge, and intention to change [27, 57], evidence that they can stimulate behavioural change is less convincing [68]. The messages through mass-media campaigns along with local-level services such as community counselling, local exercise schemes, or an individual’s social network are expected to be more widely heard and acted upon by people with higher household wealth and educational attainment who are at an increased risk of being overweight and obese. Additionally, public policies, such as nutritional labelling, controlling advertisements (especially those shown to children), and implementing taxes on sweetened beverages could be helpful to reduce malnutrition. Finally, appropriate policies are required that address the double burden of under- and overnutrition in Bangladesh, and the delivery of proper, integrated, cost-effective public health interventions to targeted communities/people is necessary to address such burdens. In such a context, with limited resources and by identifying shared drivers of under- and overnutrition, policymakers may provoke ‘double-duty action’ in existing nutrition policies that may help to handle this growing double burden of malnutrition more effectively [69].

## Conclusions

This study is among the few to date that have investigated how the double burden of undernutrition and overnutrition is distributed at the community level in Bangladesh. This study indicated that, whereas underweightness persists as a significant problem, the burden of overweightness is also increasing rapidly, underscoring the importance of future research to determine the driving forces behind the increasing rates of overweightness. This study makes an important contribution by documenting the fact that the epidemics of under- and overweightness are not present within the same community. This study adds to the literature by showing that the influence of contextual environment (i.e., community wealth status) is important with regard to the nutritional well-being of women, and, in developing policies regarding the persistent and chronic problem of underweightness and the emerging problem of overweightness among women in Bangladesh, we need to consider this contextual environment. Since Bangladesh is striving towards meeting the Sustainable Development Goals (SDGs) 2.2 plan of ending all forms of malnutrition by 2030, an integrated/holistic approach is needed to address both the under- and overnutrition among these populations.
